# Plant-Based Nutrition and Type 2 Diabetes Mellitus: Prevention, Risk, Metabolic Health and the Role of Legumes, Dietary Fiber, and Red Meat

**DOI:** 10.3390/nu18162672

**Published:** 2026-08-15

**Authors:** Fabio Frigo, Peter Fasching, Helmut Brath

**Affiliations:** 1 Department of Endocrinology, Rheumatology and Acute Geriatrics, Klinik Ottakring, 1160 Vienna, Austria; peter.fasching@gesundheitsverbund.at; 2 Diabetes and Lipid Metabolism Outpatient Clinic, Gesundheitszentrum Favoriten, 1100 Vienna, Austria; 3 Faculty of Medicine, Sigmund Freud Private University, 1020 Vienna, Austria

**Keywords:** type 2 diabetes mellitus, plant-based diet, dietary patterns, legumes, dietary fiber, red meat, insulin resistance, gut microbiota

## Abstract

**Background**: Type 2 diabetes mellitus (T2D) is one of the fastest-growing chronic diseases worldwide, affecting an estimated 589 million adults in 2024, with projections rising to 853 million by 2050. Dietary modification is a cornerstone of both T2D prevention and management. Among the various dietary approaches, plant-based dietary patterns have gained increasing attention; however, uncertainty remains regarding the optimal dietary composition and the contribution of specific food groups. **Methods**: This narrative review summarizes the current evidence on plant-based dietary patterns and their association with T2D prevention and metabolic control, with a particular focus on legumes, dietary fiber, and red and processed meat. Evidence from prospective cohort studies, randomized controlled trials, and meta-analyses was evaluated. **Results**: Higher adherence to healthy plant-based dietary patterns was consistently associated with up to a 30% lower risk of developing T2D, improved insulin sensitivity, and approximately a 0.30–0.47 percentage-point reduction in glycated hemoglobin (HbA_1c_). Greater consumption of legumes and dietary fiber was linked to improved glycemic control, enhanced but heterogeneous modulation of gut microbiota composition, reduced low-grade inflammation, and beneficial effects on body weight and lipid metabolism. In contrast, higher intake of processed meat and unprocessed red meat was consistently associated with an increased risk of T2D. Importantly, unhealthy plant-based diets characterized by refined grains, sugar-sweetened beverages, and highly processed foods provided little metabolic benefit and may even increase diabetes risk by up to 13%. The interpretation of the available evidence is limited by the predominance of observational studies, heterogeneous definitions of plant-based dietary patterns, self-reported dietary assessments, and the limited availability of long-term randomized controlled trials. **Conclusions**: Current evidence supports healthy plant-based dietary patterns as an effective strategy for the prevention and management of T2D. Dietary patterns emphasizing whole plant foods, including legumes, whole grains, fruits, vegetables, nuts, and seeds, while limiting processed and excessive red meat intake, are associated with improved metabolic health. Future high-quality randomized controlled trials with standardized dietary definitions are needed to further define the optimal composition of plant-based diets for diabetes prevention and treatment.

## 1. Introduction

Type 2 diabetes mellitus (T2D) is one of the most significant global public health challenges of the 21st century. Its prevalence continues to increase worldwide, largely driven by sedentary lifestyles, excess body weight, population aging, and substantial changes in dietary habits [1,2]. In 2024, an estimated 589 million adults between 20–79 worldwide had diabetes, with projections indicating this number will exceed 853 million by 2050 [2]. Type 2 diabetes is the most common form of diabetes, accounting for approximately 90% of all cases, representing a substantial burden on healthcare systems worldwide [1,2]. The growing global incidence of T2D is strongly associated with the increasing prevalence of obesity and is further driven by rapid economic development, urbanization, sedentary lifestyles, and unhealthy dietary patterns [2,3].

Beyond chronic hyperglycemia, T2D is characterized by insulin resistance in skeletal muscle and liver, progressive β-cell dysfunction, chronic low-grade inflammation, dyslipidemia, and ectopic fat accumulation [3,4,5]. The pathophysiology is characterized by insulin resistance and initial hyperinsulinemia, followed by progressive decline in the capacity of pancreatic β-cells to produce insulin, with 40–80% of β-cell function already lost at diagnosis of T2D [3]. Contemporary models recognize additional pathophysiological factors including incretin dysregulation, dysbiosis in gut microbiota, immune dysregulation, and islet amyloid polypeptide deposition [3,6,7]. These metabolic disturbances substantially increase the risk of cardiovascular disease, chronic kidney disease and premature death, putting an ever-growing strain on healthcare systems [3,8].

Lifestyle modification is considered a cornerstone of both prevention and treatment of T2D, with nutrition playing a central role [3,9]. Over recent decades, nutritional science has increasingly shifted away from a reductionist focus on single nutrients toward the evaluation of whole dietary patterns and food groups. This paradigm shift reflects the recognition that foods are consumed in complex combinations and that their metabolic effects arise from interactions between macronutrients, micronutrients, bioactive compounds, and dietary structure [10,11].

Accordingly, this review first summarizes the evidence on overall plant-based dietary patterns and subsequently examines key dietary components, including legumes, dietary fiber, and red and processed meat, as well as potential biological mechanisms through which they may influence metabolic health and the prevention and management of T2D.

## 2. Materials and Methods

A narrative literature review was performed to summarize and critically review the current evidence regarding the association between plant-based dietary patterns, specific food groups, and the prevention and management of T2D. Relevant literature was identified through searches of PubMed/MEDLINE as well as Google Scholar and by manually screening the reference lists of eligible articles, systematic reviews, meta-analyses, and international guidelines.

The literature search used combinations of keywords and Medical Subject Headings (MeSH) related to “type 2 diabetes mellitus”, “plant-based diet”, “vegetarian diet”, “vegan diet”, “legumes”, “dietary fiber”, “red meat”, “processed meat”, “glycemic control”, “insulin resistance”, and “HbA_1c_”.

**Figure 1:**
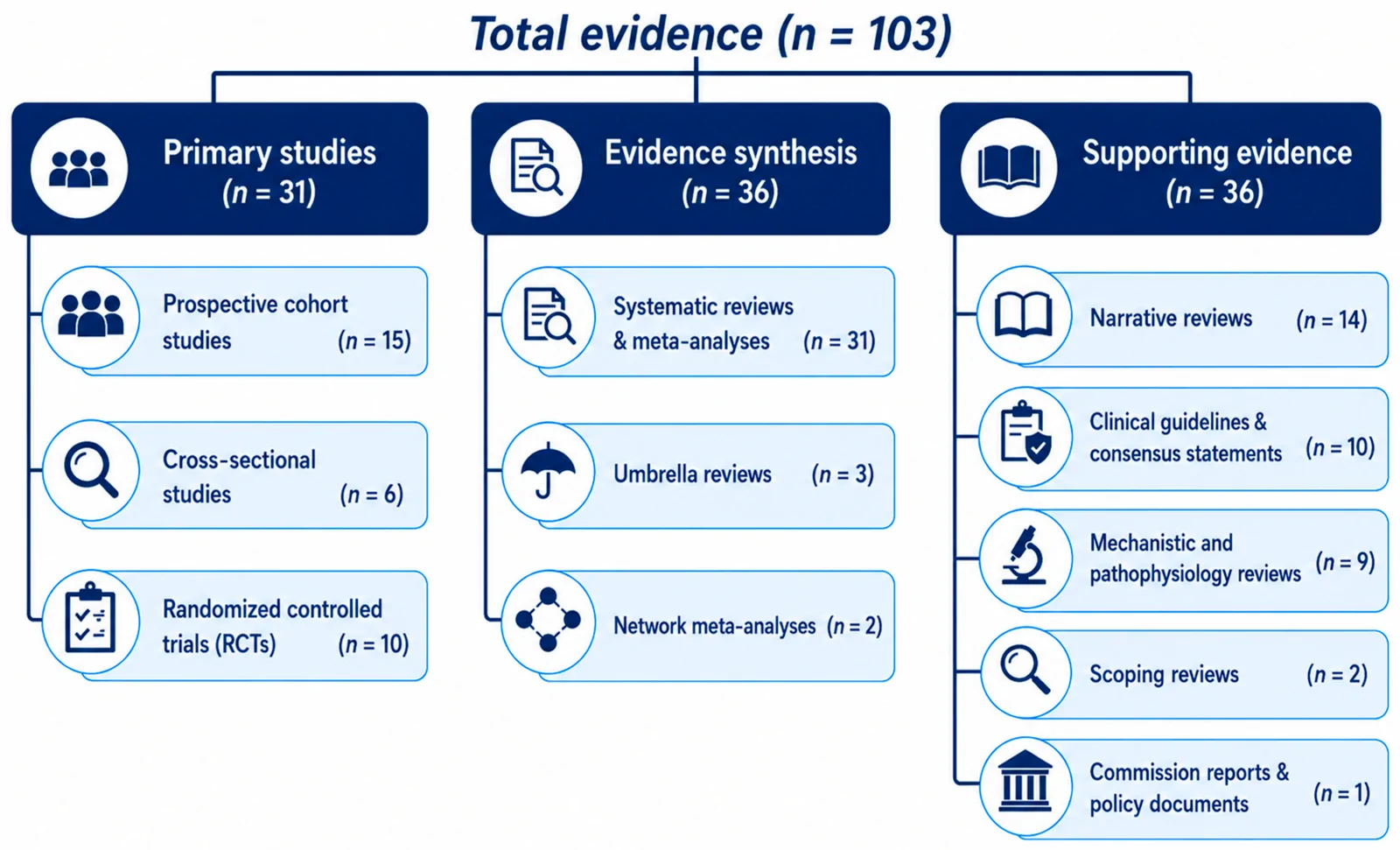
Schematic overview of the evidence included in this narrative review. Created by the authors based on the cited literature.

Peer-reviewed articles published in English or German between 2006 and 2026 were considered eligible, with particular focus placed on evidence published during the past 10 years. Priority was given to systematic reviews, meta-analyses, randomized controlled trials, and large prospective cohort studies. Narrative reviews were included selectively to provide contextual background and mechanistic insights where appropriate. All the included evidence is summarized in Figure 1.

Studies involving adults with T2D or individuals at increased risk of T2D were included. Studies focusing exclusively on type 1 diabetes, gestational diabetes, or pediatric populations were excluded unless they provided mechanistic insights relevant to T2D pathophysiology. No additional exclusion criteria regarding comorbidities, medication use, or alcohol consumption were applied.

Relevant information regarding study design, study population, dietary exposure definitions, and metabolic outcomes was extracted and synthesized narratively. Owing to the considerable heterogeneity of study designs, dietary assessment methods, definitions and scoring systems of plant-based dietary patterns, and reported outcome measures, a formal quantitative synthesis was not considered appropriate. Furthermore, most observational studies relied on self-reported food frequency questionnaires, which are susceptible to measurement error and misclassification, particularly because repeated dietary assessments were rarely performed. This represents an important limitation of the available evidence, as measurement error and changes in dietary habits over time may have influenced the observed associations.

Generative artificial intelligence (AI) was used for language editing and to provide examples of possible visual presentation formats. The final figures and tables were created and verified by the authors. All literature screening, data extraction, evidence synthesis, interpretation, and final verification of the content were performed by the authors.

## 3. Evidence on Plant-Based Dietary Patterns, Metabolic Health and Type 2 Diabetes Mellitus

### 3.1. Dietary Patterns and Metabolic Health

Western dietary patterns are characterized by high intakes of red and processed meat, refined carbohydrates, sugar-sweetened beverages, and ultra-processed foods. They have consistently been associated with obesity, insulin resistance, metabolic syndrome, and increased risk of T2D [11,12,13,14]. Consistently, unhealthy Western-style dietary patterns characterized by high intakes of sweets, refined grains, and added sugars significantly elevate T2D risk. In a large prospective cohort, this was associated with an 11% higher T2D incidence. Similarly, cross-sectional data by Pestoni et al. demonstrated that strong adherence to a Western pattern compared to a prudent diet was associated with higher odds of prediabetes and both diagnosed and undiagnosed T2D [12,14]. Owing to this cross-sectional study design, these findings reflect associations and not causation.

In contrast, dietary patterns emphasizing whole, minimally processed plant foods, such as the Mediterranean, DASH, and vegetarian/vegan diets, are consistently associated with improved metabolic health. Meta-analyses of randomized controlled trials demonstrate that these patterns significantly improve glycemic control, showing reductions in HbA_1c_ of 0.39 percentage points for the Mediterranean diet, 0.53 percentage points for the DASH diet, and 0.36–0.40 percentage points for vegetarian/vegan diets compared with control diets, alongside favorable lipid profiles and reduced cardiometabolic risk [10,11,15,16,17].

Among these, the Mediterranean diet is widely recognized as a premier approach for type 2 diabetes prevention and management. Beyond glycemic control, its high adherence is associated with a 30% reduced risk of developing T2D (supported by the Prevención con Dieta Mediterránea (PREDIMED) trial), alongside improvements in BMI (−0.29 kg/m^2^), LDL-cholesterol (−9.3 mg/dL) and blood pressure (−1.45 mmHg). These benefits are largely attributed to its rich content of monounsaturated fatty acids (primarily from olive oil), fibers, and polyphenols, which enhance insulin sensitivity and inhibit inflammatory processes [18,19,20,21,22].

More broadly, large-scale evidence confirms the preventive power of high dietary quality over individual nutrients alone: adherence to the Mediterranean, DASH, or Alternative Healthy Eating Index (AHEI) patterns reduces incident diabetes risk by up to 21%, while exploratory plant-rich patterns yield a 16% risk reduction [10,11]. Dose-response modelling suggested that an optimal intake of protective food groups could reduce T2D risk by even up to 42% [23].

Despite broad agreement on the importance of diet quality, nutritional recommendations for people with or at risk of T2D remain heterogeneous. National and international dietary guidelines vary widely in scope, level of detail, and emphasis on specific macronutrients or food groups [9,24,25,26]. According to the American Diabetes Association and the European Association for the Study of Diabetes, no single macronutrient ratio is universally optimal for people with T2D [9,27]. While most international guidelines provide comprehensive evidence-based recommendations, the recently updated Dietary Guidelines for Americans (2025–2030) adopted a substantially more concise format, offering less detailed guidance on dietary composition and food quality. In addition, these updated guidelines place a greater emphasis on animal-derived foods, representing a shift away from the stronger focus on plant-based dietary patterns seen in most other contemporary dietary recommendations [28]. Simplification obscures the biological complexity of diet-metabolism interactions, contributing to ongoing controversy and public confusion regarding optimal dietary strategies.

### 3.2. Plant-Based Dietary Patterns and Diabetes Risk

In this context, plant-based dietary patterns have received increasing attention as a strategy for the prevention and management of T2D. “Plant-based nutrition” is an umbrella term encompassing a variety of dietary approaches in which plant-derived foods, including vegan, vegetarian, lacto-ovo-vegetarian, and flexitarian diets in which plant-derived foods, including vegetables, fruits, whole grains, legumes, nuts, and seeds, constitute the primary source of energy and nutrients. Importantly, plant-based dietary patterns do not necessarily exclude animal-derived foods. Flexitarian diets and the Planetary Health Diet (PHD) emphasize a predominantly plant-based dietary pattern while allowing moderate amounts of sustainably sourced animal-source foods, thereby distinguishing them from strictly vegetarian or vegan diets [29].

**Figure 2:**
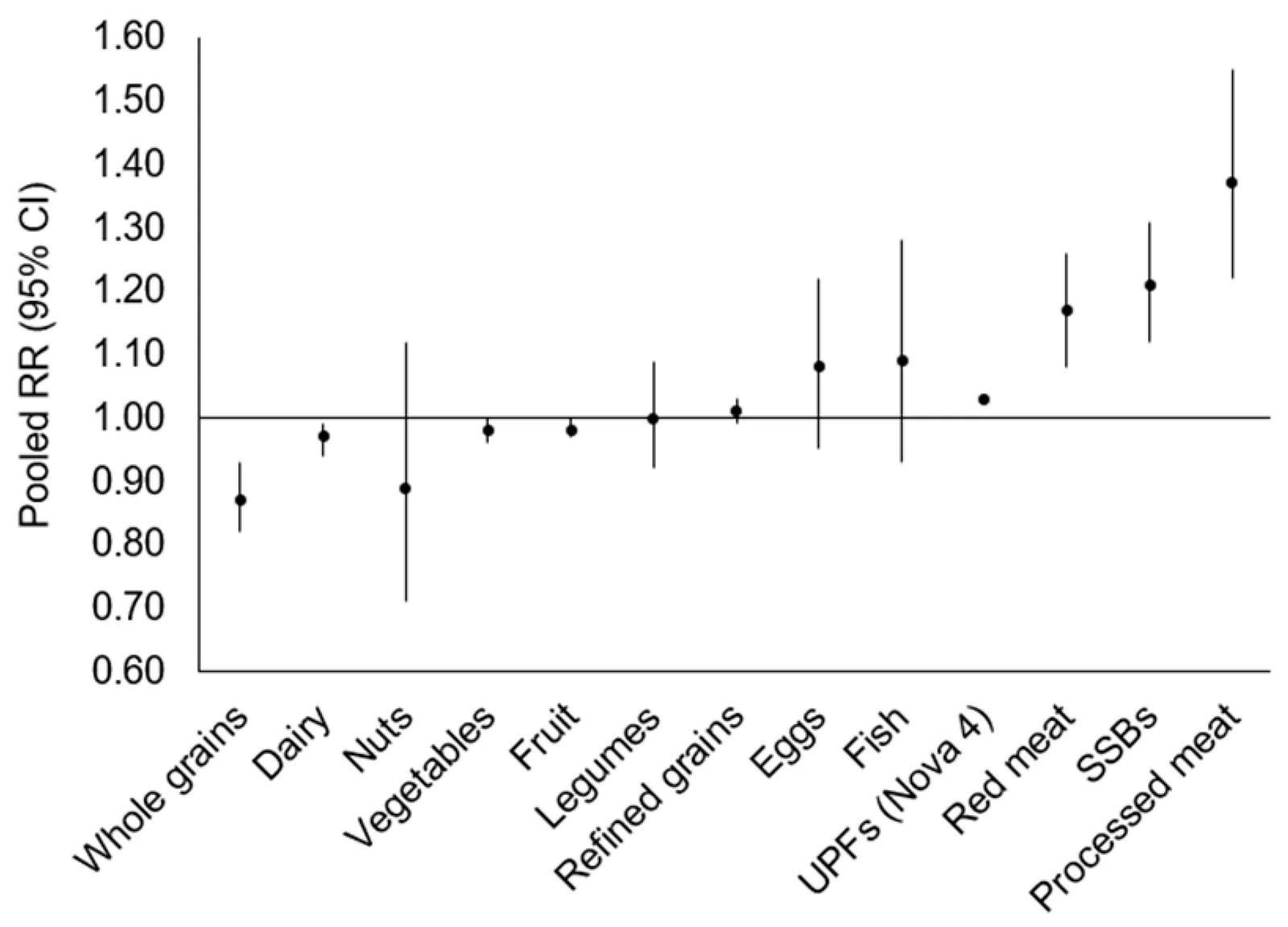
Pooled relative risk (RR) estimates for incident type 2 diabetes according to intake of selected foods and food groups with varying degrees of processing, including ultra-processed foods (UPFs). Error bars represent 95% confidence intervals. The estimates are based on previously published random-effects meta-analyses of prospective cohort studies using multivariable-adjusted models. In the original figure, estimates for whole grains, dairy, nuts, vegetables, fruit, legumes, refined grains, eggs, fish, red meat, sugar-sweetened beverages (SSBs), and processed meat were derived from Schwingshackl et al., whereas the estimate for UPFs was derived from Chen et al. [23,37]. The intake amounts and exposure definitions underlying the risk estimates differ across food groups and source meta-analyses; therefore, the shared axis is intended for visual overview only and does not imply direct comparability between exposures. Reproduced unchanged from Mendoza et al. under license from Springer Nature [33].

Evidence from large prospective cohort studies, systematic reviews, and meta-analyses consistently demonstrates that greater adherence to plant-based dietary patterns is associated with a lower risk of incident T2D [29,30,31,32]. Figure 2 provides a visual overview of pooled relative risk estimates for incident T2D across selected foods and food groups, as reproduced from Mendoza et al. [33]. A meta-analysis of nine studies including 307,099 participants and 23,544 incident T2D cases found that adherence to plant-based dietary patterns was associated with a 23% lower relative risk of developing type 2 diabetes. Moreover, prospective cohort studies indicate that declining adherence to plant-based dietary patterns over time is associated with an increased risk of developing the disease [30,32]. Comparable findings have also been reported for the EAT-Lancet reference diet, with participants showing the highest adherence exhibiting a 10–43% lower risk of incident T2D than those with the lowest adherence, although the magnitude of the association varied according to cohort characteristics, scoring systems, and adjustment for BMI [34,35,36].

Importantly, recent research differentiates between healthful (hPDI) and unhealthful plant-based diets (uPDI) [30,31,38,39]. While diets rich in whole grains, legumes, fruits, vegetables, and nuts (hPDI) are associated with substantial reductions in diabetes risk, plant-based diets dominated by refined grains, added sugars, and sugar-sweetened beverages (uPDI) confer little benefit or even increase metabolic risk [30,31,39]. This distinction is particularly relevant regarding ultra-processed plant-based foods, including confectionery and many commercially available meat substitutes. These products are frequently characterized by high amounts of refined carbohydrates, sodium, added sugars, and food additives, while providing low amounts of dietary fiber and intact food matrices. Consequently, high consumption of these ultra-processed options can attenuate or even negate the metabolic benefits typically associated with healthy plant-based eating. Ultimately, these findings underscore that food quality and the degree of food processing, rather than the mere exclusion of animal products, are the critical determinants of metabolic outcomes [30,31,40].

Randomized controlled trials and meta-analyses provide mechanistic evidence supporting the beneficial metabolic effects of plant-based dietary patterns in T2D [30,41,42,43]. Intervention studies demonstrate that vegetarian and low-fat plant-based diets are associated with improvements in insulin resistance, β-cell function, oxidative stress, and inflammatory biomarkers, effects largely mediated by reductions in body weight, visceral and intramyocellular fat, and subsequent improvements in insulin signaling pathways [41,42,43]. Together, these findings suggest that high-fiber, low-saturated fat plant-based diets may exert their antidiabetic effects through a combination of lipid redistribution, attenuation of chronic low-grade inflammation, and improved postprandial glucose regulation [30,41,42,43]. Among the various components of plant-based dietary patterns, dietary fiber and legumes appear to play a particularly important mechanistic role in mediating metabolic benefits, as summarized in Table 1.

### 3.3. Role of Legumes, Dietary Fiber and the Gut Microbiota

Among plant-based food groups, legumes and dietary fiber appear to play a particularly important role in glucose metabolism and insulin sensitivity [55,57,61,66]. Different types of dietary fiber influence glucose metabolism through complementary mechanisms. Soluble, gel-forming fibers increase intestinal viscosity, delay gastric emptying, and slow glucose diffusion, thereby attenuating postprandial glucose and insulin excursions [55,56,67]. In contrast, insoluble cereal fibers have been most consistently associated with a lower risk of incident T2D in prospective cohort studies and may improve insulin sensitivity, although findings from randomized trials remain less consistent [68]. Specifically, whole grain consumption (per 30 g/day increment) is associated with a 13% reduction in T2D risk, and cereal fiber intake (per 10 g/day increment) with a 25% risk reduction [15]. Resistant starch escapes digestion in the small intestine and is fermented by the gut microbiota into short-chain fatty acids (SCFAs), which may stimulate glucagon-like peptide-1 (GLP-1) secretion and enhance glucose-dependent insulin release [69]. Similarly, low-glycemic-index carbohydrate sources reduce postprandial glucose excursions and have been associated with modest improvements in HbA_1c_ and fasting glucose [70]. Together, these complementary mechanisms are likely to contribute to the favorable metabolic effects of high-fiber plant-based dietary patterns, although their relative contribution in free-living populations remains incompletely understood [68].

Legumes, as a rich source of soluble and insoluble fiber, plant protein, and bioactive compounds, have been consistently linked to improved insulin sensitivity and cardiometabolic health [56,57,66]. A meta-analysis of prospective cohort studies found that legume consumption of ≥4 servings (100 g each) per week was inversely associated with CHD risk (RR 0.86, 95% CI 0.78–0.94) [48]. Furthermore, a meta-analysis of 41 randomized controlled trials demonstrated that dietary legumes significantly improved glycemic control, predominantly by reducing postprandial blood glucose and insulin excursions due to their low glycemic index [56]. Beyond these immediate postprandial effects, the high content of fermentable fibers in legumes is thought to modulate the gut microbiota, promoting the growth of bacteria that produce SCFAs. This shift in microbial diversity and functional capacity contributes to improved glucose homeostasis, enhanced insulin sensitivity, and reduced low-grade systemic inflammation [71,72,73,74]. However, recent systematic evidence notes that while plant-based and Mediterranean interventions consistently modulate gut microbial composition, their subsequent effects on HbA_1c_ and fasting glucose remain heterogeneous across studies. In contrast to these fiber-rich patterns, Western dietary patterns induce dysbiosis and impair gut barrier function, thereby aggravating insulin resistance [75,76,77,78]. Ultimately, these distinct pathways align with current clinical guidelines that prioritize minimally processed, fiber-rich carbohydrate sources over refined carbohydrates [25,79].

### 3.4. Red Meat Consumption and Metabolic Risk

In contrast, higher consumption of red and processed meat has been associated with increased risk of T2D and adverse cardiometabolic outcomes [15,44,46,49,50,80]. The detrimental metabolic effects of meat consumption are well-documented across large-scale epidemiological data. A massive observational analysis of 1.97 million adults quantified this risk, showing that each additional 50 g/day of processed meat was associated with a 15% increased risk of T2D, while 100 g/day of unprocessed red meat raised the risk by 10% [49,55]. Compounding these findings, an umbrella review of meta-analyses confirmed a dose-dependent relationship, reporting even steeper risk escalations: a 17% higher T2D risk per 100 g/day of unprocessed red meat, and a 37% increase in risk for every additional 50 g/day of processed meat [15,44]. Consistent with these findings, consumption of risk-increasing foods (red meat, processed meat, and sugar-sweetened beverages) at levels associated with the highest risk was associated with an approximately threefold higher T2D risk compared to non-consumption [23].

Proposed mechanisms include higher intake of saturated fat, heme iron, nitrates, and advanced glycation end products, as well as pro-inflammatory metabolic byproducts [46,50]. After accounting for heme iron intake, the association between red meat consumption and T2D remained statistically significant. In contrast, heme iron intake continued to be independently associated with an increased risk of T2D after adjustment for red meat consumption [50]. While the Dietary Guidelines for Americans 2025–2030 place renewed emphasis on animal-source foods, including red meat and full-fat dairy, concerns remain regarding the long-term implications of higher consumption of these foods. In particular, greater intake of red and processed meat has also been associated with substantially greater greenhouse gas emissions, land use, and freshwater consumption compared with plant-forward dietary patterns [28,81,82,83].

The evidence base suggests that the metabolic effects of animal-derived foods are context-dependent and influenced by overall dietary patterns, food processing, and substitution effects [49]. Substituting processed meat with either unprocessed red meat or poultry was associated with a 7–10% reduced risk of T2D [49]. Overall dietary quality and the source of dietary protein appear to be more important determinants of metabolic health than total protein intake alone, and increasing protein intake without consideration of food quality may yield heterogeneous effects [10,30].

The ADA Standards of Care 2026 note that replacing animal proteins with plant proteins specifically leads to small improvements in HbA_1c_ and fasting glucose [79]. These differences are thought to be due to the accompanying nutrients and bioactive compounds found in plant proteins, such as dietary fiber, unsaturated fatty acids and phytochemicals, which are absent in animal proteins [51,79].

### 3.5. Low-Carbohydrate Approaches and Dietary Controversies

Low-carbohydrate and ketogenic dietary approaches have gained popularity due to their capacity to induce rapid improvements in glycemic control, insulin sensitivity and weight loss [9,58,79,84,85]. Systematic reviews and clinical studies report short-term reductions in HbA_1c_ and fasting glucose; a meta-analysis demonstrated that very low-carbohydrate ketogenic diets resulted in greater decreases in HbA_1c_ after 3 months and after 6 months compared with control diets [59,84,86]. Network meta-analyses indicate that ketogenic, low-carbohydrate, and Mediterranean diets demonstrate promising effects for controlling HbA_1c_ and fasting glucose [58].

However, concerns remain regarding long-term adherence, cardiovascular safety, fiber inadequacy, and sustainability, particularly in real-world settings where lifestyle disruptions, weight gain, physical inactivity, psychological stress, and insufficient sleep have been shown to adversely affect glycemic control in people with T2D [9,79,85,87,88]. The greater glycemic benefits of low-carbohydrate diets at 3 and 6 months are not evident with longer follow-up [9,86,87,89]. A meta-analysis of six RCTs with ≥12 months follow-up found that carbohydrate-restricted eating patterns improved lipid profiles but did not significantly improve glycemic control [79]. Additionally, studies on low-carbohydrate eating patterns frequently report challenges regarding long-term adherence and sustainability. Interpretation of the evidence is further complicated by substantial variation in the definitions of low-carbohydrate diets and by the confounding effects of weight loss, which may independently influence metabolic outcomes [79,86,89,90].

Importantly, low-carbohydrate diets in practice frequently default to higher intakes of fat and protein, often through increased consumption of red and processed meat as primary macronutrient replacements, which may diminish potential glycemic benefits. The ketogenic diet is the most restrictive form of carbohydrate restriction, typically limiting carbohydrate intake to less than 50 g/day (or <10% of total energy) to achieve and maintain nutritional ketosis, with the majority of energy derived from fat and a moderate intake of protein [85]. Prospective data demonstrated that animal-based low-carbohydrate diets were associated with a 39% higher risk of T2D, whereas healthy plant-based low-carbohydrate diets emphasizing vegetable protein and unsaturated fats were associated with a 16% lower risk [53]. A potential limitation of low-carbohydrate diets is the reduced intake of dietary fiber, as many carbohydrate-rich foods are restricted. Nevertheless, adequate fiber intake can be partially maintained through the careful selection of low-carbohydrate vegetables and other fiber-rich plant foods [85].

A recent network meta-analysis of randomized controlled trials ranked Mediterranean, moderate-carbohydrate, and low-glycemic-index diets among the most effective dietary strategies for improving HbA_1c_ and fasting glucose concentrations. Although vegetarian and vegan diets also produced significant reductions in HbA_1c_ compared with control diets, their effects were generally smaller than those observed with these dietary patterns [58].

These ongoing debates are reflected in the variability, and sometimes apparent contradictions, of dietary guidelines. This further highlights the need for a nuanced interpretation of the evidence, rather than the provision of simple dietary prescriptions [9,25,79].

## 4. Discussion

The present review provides an integrated overview of the current evidence on plant-based dietary patterns and their role in the prevention and management of T2D, with particular emphasis on dietary fiber, legumes, red and processed meat, and their proposed biological mechanisms. Overall, evidence from prospective cohort studies and randomized trials consistently supports that higher adherence to healthful plant-based dietary patterns (hPDI), rich in whole grains, legumes, fruits, vegetables, and nuts, is associated with up to a 30% lower risk of T2D and improved glycemic control [30,31,91].

Importantly, these benefits depend heavily on overall food quality and the degree of processing rather than the mere exclusion of animal products [30,31]. Plant-based diets dominated by refined carbohydrates and ultra-processed foods (uPDI) confer little metabolic benefit and may even increase T2D risk by up to 13% [30,31,91].

Under the NOVA food classification system (NOVA), certain plant-based alternatives (for example: oat drinks, tofu, and meat substitutes) are classified as ultra-processed; however, prospective evidence suggests they are not associated with the increased cardiometabolic risk observed for ultra-processed animal-source foods or sugar-sweetened beverages [92].

Several complementary biological mechanisms plausibly explain these findings [30,93,94]. Dietary fiber represents a central protective component by improving satiety, reducing postprandial glycemic excursions, and contributing to body weight regulation [95,96,97]. Furthermore, fermentable fibers promote the microbial production of SCFAs, which enhance gut barrier integrity, reduce systemic inflammation, and improve insulin sensitivity [95,97,98].

By stimulating intestinal L-cells, SCFAs enhance the release of GLP-1 and peptide YY, thereby promoting insulin secretion, suppressing glucagon release, slowing gastric emptying, and increasing satiety [95,98]. A randomized clinical trial suggests that diets rich in fiber selectively enrich SCFA-producing bacterial strains, which may directly mediate improvements in HbA_1c_ via enhanced GLP-1 production [63].

Legumes deserve particular attention because they combine several metabolically favorable properties, including high fiber density, low glycemic index, plant-derived protein and resistant starch [66]. Meta-analytic data from 41 randomized controlled trials indicate that legumes significantly improve glycemic control by reducing postprandial glucose and insulin excursions [56]. Furthermore, clinical evidence demonstrates that replacing one daily serving of red meat with legumes as part of a hypocaloric DASH diet significantly enhances fasting plasma glucose reductions (16 mg/dL versus 7 mg/dL) and HOMA-IR improvements (1.2 versus 0.7 units) compared to a standard DASH diet [64]. Cross-sectional insights suggest these effects are partly mediated through the enrichment of beneficial butyrate-producing gut microbes [65]. Conversely, red and processed meat consumption is consistently linked to adverse metabolic outcomes through pathways involving saturated fats, nitrosamines, advanced glycation end products, and heme iron exposure [49,50,93,94,99]. The largest federated individual-participant meta-analysis to date (1.97 million adults) robustly confirmed this positive association with incident T2D [49]. Heme iron intake has been independently associated with a 14% higher risk of T2D after adjustment for meat intake. Mechanistically, heme iron has been hypothesized to promote oxidative stress through Fenton-reaction-mediated pathways, which may contribute to impaired insulin signaling and pancreatic β-cell dysfunction [29,50]. However, animal-based foods are metabolically heterogeneous. For example, fermented dairy products may have more favorable cardiometabolic effects than processed meats, highlighting the importance of a differentiated, food-based approach to dietary recommendations [83,99].

Current evidence also suggests that the source and quality of macronutrients are likely more relevant than absolute macronutrient quantity alone [13,79,100]. While low-carbohydrate or ketogenic diets may improve HbA_1c_ and body weight in the short term, their long-term superiority remains uncertain due to adherence challenges and potential risks regarding gut health and cardiovascular safety [13,85,100,101]. Systematic reviews confirm that vegetarian, vegan, and Mediterranean dietary patterns significantly reduce HbA_1c_ (by approximately 0.3–0.4 percentage points), with the Mediterranean pattern showing the strongest evidence for weight loss, glycemic durability, and a delayed requirement for diabetes medication [100,101].

Consequently, evidence-based guidelines favor individualized dietary counseling that prioritizes long-term adherence, food quality, and patient preferences over rigid macronutrient targets. Successful implementation requires interdisciplinary collaboration and must account for socioeconomic disadvantages, health literacy, food insecurity, and language barriers through culturally adaptable, practical dietary strategies [102].

Beyond metabolic health, plant-forward dietary patterns may also provide important environmental benefits. Diets characterized by lower consumption of animal products are associated with substantially reduced greenhouse gas emissions, freshwater use, land use and environmental burden [9,79,80,82,103]. Therefore, plant-based dietary strategies may offer a unique opportunity to simultaneously address cardiometabolic health and environmental sustainability. Nevertheless, it is important to recognize that not all plant-based diets are environmentally or metabolically equivalent, particularly when characterized by high levels of ultra-processed foods [28,29,31,91]. Approximately 30% of global greenhouse gas emissions are attributable to food systems, with animal-based food production contributing the largest share of agricultural emissions [83]. Currently, 37% of the global land area is used for agricultural production, of which two-thirds is grazing land predominantly for animals [83].

Despite the consistency of the available evidence, several methodological limitations should be acknowledged. Most evidence originates from prospective cohort studies, which remain susceptible to residual confounding and healthy-user bias; individuals adhering to healthful plant-based diets frequently engage in other health-promoting behaviors, such as higher physical activity and lower smoking prevalence [13,30]. Furthermore, reliance on self-reported food frequency questionnaires introduces recall errors, measurement bias, and dietary misclassification, failing to fully capture dietary changes over time. Many randomized controlled trials are additionally limited by small sample sizes, lack of blinding, declining adherence, and short follow-up periods, which restrict long-term conclusions regarding efficacy and sustainability [13,17,99,100,101]. Finally, due to the narrative nature of this review, study selection and evidence synthesis were not guided by a predefined protocol or formal risk-of-bias assessment, meaning these findings represent a qualitative synthesis rather than a comprehensive systematic evaluation.

Future research should focus on standardized long-term intervention studies conducted in real-world settings, with improved characterization of food quality and degree of processing [11,13,17]. Additional research should focus on food substitution patterns, personalized dietary interventions, gut microbiota-related mechanisms, and implementation strategies aimed at improving long-term adherence [11,13,96]. Integration of clinical, metabolic and environmental outcomes may further strengthen future dietary recommendations for T2D prevention and management [45].

## 5. Conclusions

Overall, the current body of evidence supports healthful plant-based dietary patterns as an effective strategy for both the prevention and management of T2D. The greatest metabolic benefits are observed with dietary patterns rich in minimally processed plant foods, including whole grains, legumes, fruits, vegetables, nuts, and seeds, while limiting processed meat and excessive consumption of red meat. Importantly, the available evidence indicates that overall food quality, degree of processing, and food substitution patterns are more influential determinants of metabolic health than isolated macronutrient composition alone. Future dietary recommendations should therefore move beyond reductionist debates focusing on carbohydrates or plant- versus animal-based diets and instead promote sustainable, individualized, culturally adaptable, and food-based dietary patterns that support long-term adherence, metabolic health, and environmental sustainability.

## Figures and Tables

**Table 1:** Dietary Patterns, Food Groups, and Type 2 Diabetes: Summary of Evidence.

Category	Study	Study Design/Population	Dietary Exposure/Intervention	Main Metabolic Findings
**Umbrella reviews**	Neuenschwander et al., 2019 [15]	Umbrella review of meta-analyses, 53 publications, 153 risk estimates	Whole grains, dietary fiber, red/processed meat, SSB	Whole grains (+30 g/d): HR 0.87 (0.82–0.93); cereal fiber (+10 g/d): HR 0.75 (0.65–0.86); red meat (+100 g/d): HR 1.17 (1.08–1.26); processed meat (+50 g/d): HR 1.37 (1.22–1.54); SSB (+1 serving/d): HR 1.26 (1.11–1.43)—all high GRADE evidence
	Zhang et al., 2023 [44]	Umbrella review, 40 meta-analyses	Red and processed meat consumption	Red meat (+100 g/d): +17% T2D risk; processed meat (+50 g/d): RR 1.37 (1.22–1.55) for T2D
	Schwingshackl et al., 2017 [23]	SR & meta-analysis, 12 food groups, prospective studies	Whole grains, fruit, dairy, red/processed meat, SSB	Risk-reducing foods at optimal intake (modelling): −42% T2D risk; risk-increasing foods: 3-fold T2D risk. High evidence for processed/red meat, whole grains, SSB
	Banjarnahor et al., 2025 [45]	Umbrella review, 67 articles, 46 meta-analyses, 13 food groups	Whole grains, fruit, vegetables, nuts, legumes, meat, SSB	Whole grains: ↓ T2D risk (moderate evidence); fruit/vegetables: ↓ T2D risk (moderate); processed meat: ↑ T2D risk (high evidence); red meat: ↑ (moderate); SSB: ↑ (moderate)
**Systematic reviews & meta-analyses of prospective studies**	Jannasch et al., 2017 [11]	Meta-analysis, 48 articles, 16 cohorts	Mediterranean diet, DASH, AHEI dietary patterns	Mediterranean: RR 0.87 (0.82–0.93); DASH: RR 0.81 (0.72–0.92); AHEI: RR 0.79 (0.69–0.90); “mainly unhealthy” pattern: RR 1.44 (1.27–1.62); “mainly healthy”: RR 0.84 (0.77–0.91)
	Wallerer et al., 2025 [21]	Dose-response meta-analysis, 24 cohorts + 1 RCT, 991,878 participants, 68,325 T2D cases	Mediterranean diet adherence	HR per 2-point increment: 0.92 (0.90–0.94), moderate GRADE evidence; RCT (PREDIMED): HR 0.75 (0.56–1.01)
	Qian et al., 2019 [30]	Meta-analysis, 9 studies, 307,099 participants, 23,544 T2D cases	Plant-based dietary patterns	Highest vs. lowest adherence: RR 0.77 (0.71–0.84); healthy plant-based patterns: RR 0.70 (0.62–0.79)
	Murciano et al., 2025 [31]	Dose-response meta-analysis, 36 studies (1999–2025)	Healthy and unhealthy plant-based diets	Vegan diet: RR 0.65 (0.42–1.00); lacto-ovo-vegetarian: RR 0.68 (0.57–0.82); hPDI: RR 0.76 (0.69–0.82); uPDI: RR 1.13 (0.98–1.30)
	Nikparast et al., 2025 [39]	Dose-response meta-analysis, 16 studies, 721,012 participants	Plant-based diet indices (O-PDI, hPDI, uPDI)	O-PDI: ES 0.86 (0.82–0.90), −14% T2D risk; hPDI: ES 0.81 (0.75–0.88), −19%; uPDI: ES 1.10 (1.04–1.16), +10%
	Shi et al., 2023 [46]	SR & meta-analysis, 27 observational studies (1,760,774 participants for diabetes)	Unprocessed and processed red meat	Unprocessed red meat (100 g/d): HR 1.11 (1.05–1.16) for CVD; processed meat (50 g/d): HR 1.26 (1.18–1.35) for CVD; both positively associated with T2D and GDM; stronger association in Western settings
	Fotouhi Ardakani et al., 2024 [47]	Dose-response meta-analysis, 16 cohorts, 615,125 participants, 52,342 T2D cases	Total, animal, and plant protein intake	Animal protein (highest vs. lowest): RR 1.18 (1.09–1.27); per 20 g increase: +7% T2D risk; plant protein: RR 0.98 (n.s.); substitution animal→plant (per 20 g): RR 0.80 (0.76–0.84), −20%
	Afshin et al., 2014 [48]	SR & meta-analysis, prospective studies	Nuts and legumes	Nuts (4 servings/week): T2D risk RR 0.87 (0.81–0.94); legumes: no significant association with T2D (only 2 studies); legumes and IHD: RR 0.86 (0.78–0.94); inverse association between nut consumption and the risk of incident ischemic heart disease (IHD) and diabetes, as well as between legume consumption and incident IHD.
**Federated/individual-participant meta-analyses**	Li et al., 2024 [49]	Federated meta-analysis, 1.97 million adults, >100,000 T2D cases, 31 cohorts, 20 countries	Unprocessed red meat, processed meat, poultry	Unprocessed red meat (100 g/d): HR 1.10 (1.06–1.15); processed meat (50 g/d): HR 1.15 (1.09–1.22); poultry (100 g/d): HR 1.08 (1.02–1.14)
**Prospective cohort studies**	Ahmad et al., 2020 [22]	Prospective cohort, 25,317 women, Women’s Health Study, 19.8 years follow-up	Mediterranean diet	MED score ≥6 vs. ≤3: HR 0.70 (0.62–0.79), −30% T2D risk; mediators: insulin resistance, adiposity, HDL, inflammation
	Gao et al., 2022 [12]	Prospective cohort, 120,343 UK Biobank participants, 8.4 years follow-up	Dietary patterns (Reduced Rank Regression)	Dietary pattern 1 (chocolate, butter, low-fiber bread, sugar): HR 1.09 (1.06–1.12) per z-score (BMI-adjusted); stronger effect in younger adults: HR 1.13 (1.09–1.18)
	Chen et al., 2021 [32]	3 prospective US cohorts (NHS, NHSII, HPFS), 192,567 participants, 12,627 T2D cases	Changes in plant-based diet adherence over 4 years	10% increment in PDI: HR 0.93 (0.91–0.95); 10% increment in hPDI: HR 0.91 (0.87–0.95); >10% decrease in hPDI: HR 1.23 (1.16–1.31)
	Talaei et al., 2017 [50]	Prospective cohort, 63,257 adults, Singapore Chinese Health Study, 10.9 years follow-up	Heme iron and meat intake	Red meat (Q4 vs. Q1): HR 1.23 (1.14–1.33); poultry: HR 1.15 (1.06–1.24); heme iron (independent of meat): HR 1.14 (1.02–1.28)
	Li et al., 2022 (WHI + UK Biobank) [51]	2 prospective cohorts, 108,681 + 34,616 participants, 15,842 T2D cases, 15.8 years follow-up	Animal vs. plant protein sources	Animal protein (Q5 vs. Q1): HR 1.31 (1.24–1.37); plant protein: HR 0.82 (0.78–0.86); substitution 5% energy plant→animal: HR 0.79 (0.74–0.84), −21%; mediators: hs-CRP, IL-6, leptin, SHBG
	Chen et al., 2020 (Rotterdam Study) [52]	Prospective cohort, 6822 participants, 643 T2D cases	Specific dietary protein (meat, fish, dairy, plant)	Animal protein (per 5% energy): HR 1.37 (1.26–1.49) for T2D; HR 1.35 (1.24–1.45) for prediabetes; HOMA-IR: β +0.10 (0.07–0.12); plant protein: no association with T2D
	Pestoni et al., 2021 (KORA FF4) [14]	Cross-sectional, 1305 participants, Southern Germany	Western vs. prudent dietary pattern	Western pattern: OR 1.92 (1.35–2.73) for prediabetes; OR 10.12 (4.19–24.43) for undiagnosed diabetes; OR 3.51 (1.85–6.67) for prevalent diabetes
	Liu et al., 2025 [53]	3 prospective US cohorts (NHS, NHSII, HPFS)	Low-carbohydrate diets of varying macronutrient quality	Healthy low-carb diet (plant-based, high-quality fat/protein): lower T2D risk; unhealthy low-carb diet (animal-based, refined carbs): higher T2D risk
**Systematic reviews & meta-analyses of RCTs**	Wu et al., 2025 [18]	Meta-analysis of RCTs, T2D patients	Mediterranean diet	HbA_1c_: MD −0.47 percentage points; BMI: MD −0.67 kg/m^2^; LDL: MD −0.24 mmol/L; systolic BP: MD −2.16 mmHg
	Lv et al., 2025 [16]	Meta-analysis, 9 RCTs, 681 participants	Vegetarian/vegan diets in T2D	HbA_1c_: WMD −0.36 percentage points (−0.54 to −0.19); LDL: WMD −0.16 mmol/L; BMI: WMD −0.94 kg/m^2^
	Guest et al., 2024 [17]	Meta-analysis, 7 RCTs, 770 participants	Vegetarian/vegan diets in T2D	HbA_1c_: MD −0.40 percentage points (−0.59 to −0.21), moderate GRADE evidence
	Viguiliouk et al., 2015 [54]	Meta-analysis, 9 RCTs, 418 participants	Replacement of animal protein with plant protein	HbA_1c_: MD −0.15 percentage points (not statistically significant)
	Reynolds et al., 2020 [55]	SR & meta-analysis, 42 RCTs (1789 participants) + 2 cohorts (8300 participants)	Dietary fiber and whole grains	HbA_1c_: MD −0.18 percentage points (−0.30 to −0.06); fasting glucose: MD −0.56 mmol/L; HOMA-IR: MD −1.24; total cholesterol: MD −0.34 mmol/L; mortality: 14 fewer deaths/1000 (35 vs. 19 g/d fiber)
	Sievenpiper et al., 2009 [56]	Meta-analysis, 41 RCTs (duration ≥7 days)	Non-oil-seed pulses (legumes)	Fasting glucose (alone): SMD −0.82 (−1.36 to −0.27); fasting insulin: SMD −0.49 (−0.93 to −0.04); HbA_1c_ in low-GI diets: SMD −0.28 (−0.42 to −0.14)
	Bielefeld et al., 2020 [57]	Systematic review of RCTs	Legume consumption	Improvements in fasting glucose and glycemic markers (qualitatively reported; no pooled effect estimates)
	Jing et al., 2023 [58]	Network meta-analysis of RCTs	Mediterranean, low-carb, and ketogenic diets	Mediterranean: HbA_1c_ −0.47 percentage points (−0.82 to −0.12); low-carb: HbA_1c_ −0.49 percentage points (−0.84 to −0.14); all 9 diets: HbA_1c_ reduction −0.47 to −0.82 percentage points vs. control diet
	Zhou et al., 2022 [59]	Meta-analysis of RCTs, overweight T2D patients	Ketogenic diet	Short-term improvements in HbA_1c_, fasting glucose, triglycerides, and body weight (qualitatively reported; no pooled MDs provided)
	Goldenberg et al., 2021 [60]	SR & meta-analysis, 23 RCTs, 1357 participants	Low-carbohydrate diets (<130 g/d) in T2D	Remission (HbA_1c_ < 6.5%) at 6 months: 57% vs. 31% (RD 0.32, 0.17–0.47); at 12 months: effect no longer significant; LDL increase and quality-of-life deterioration at 12 months
	Juhász et al., 2023 [61]	Network meta-analysis, 46 RCTs, 2685 patients, 16 fiber types	Soluble dietary fibers (galactomannans, β-glucans, psyllium, etc.)	Galactomannans: highest effect on HbA_1c_ (SUCRA 92.3%) and fasting glucose (SUCRA 85.9%); psyllium: highest effect on HOMA-IR (SUCRA 96.7%); β-glucans: HOMA-IR (SUCRA 73.5%)
**Randomized controlled trials**	Salas-Salvadó et al., 2014 (PREDIMED subgroup) [20]	RCT subgroup analysis, 3541 non-diabetic adults with high CV risk, 4.1 years follow-up	Mediterranean diet + EVOO vs. + nuts vs. control (low-fat)	MedDiet + EVOO: HR 0.60 (0.43–0.85), −40% T2D incidence; MedDiet + nuts: HR 0.82 (0.61–1.10, n.s.); incidence rates: 16.0 vs. 18.7 vs. 23.6/1000 person-years
	Kahleova et al., 2020 [62]	RCT, 244 overweight adults, 16 weeks	Low-fat plant-based diet vs. no change	Weight: −5.9 kg (5.0–6.7); HOMA-IR: −1.3 (−2.2 to −0.3); PREDIM: +0.9 (0.5–1.2); liver fat: −34.4%; intramyocellular fat: −10.4%
	Kahleova et al., 2011 [43]	RCT, 74 T2D patients, 24 weeks	Vegetarian vs. conventional diabetic diet (both −500 kcal/d)	Insulin sensitivity: +30% vs. +20% (*p* = 0.04); weight: −6.2 kg vs. −3.2 kg; visceral fat ↓ (*p* = 0.007); SOD ↑ (*p* < 0.001); 43% vs. 5% reduced medication
	Zhao et al., 2018 [63]	RCT, T2D patients	High-fiber diet targeting gut microbiota	Increased SCFA-producing bacteria; greater HbA_1c_ improvement with higher diversity/abundance of fiber-promoted producers; potentially mediated via GLP-1 production
	Hosseinpour-Niazi et al., 2022 [64]	RCT, 300 T2D patients, 16 weeks	Hypocaloric legume-based DASH diet vs. standard DASH	Legume-DASH: greater reduction in fasting glucose and HOMA-IR vs. standard DASH (*p*-values significant); both groups: ↓ FPG, insulin, TG, TC, LDL
**Multi-omics/observational + interventional**	Ma et al., 2026 [65]	Multi-omics study (observational + interventional data)	Legume and soybean consumption	Improved fasting glucose, insulin sensitivity, and beneficial gut microbiota modulation (qualitatively reported)

AHEI, Alternative Healthy Eating Index; BMI, Body Mass Index; BP, Blood Pressure; CI, Confidence Interval; CVD, Cardiovascular Disease; DASH, Dietary Approaches to Stop Hypertension; ES, Effect Size; EVOO, Extra Virgin Olive Oil; FPG, Fasting Plasma Glucose; GDM, Gestational Diabetes Mellitus; GLP-1, Glucagon-Like Peptide-1; GRADE, Grading of Recommendations Assessment, Development and Evaluation; HbA_1c_, Glycated Hemoglobin A1c; HDL, High-Density Lipoprotein; HOMA-IR, Homeostatic Model Assessment for Insulin Resistance; HPFS, Health Professionals Follow-up Study; HR, Hazard Ratio; hPDI, Healthy Plant-Based Diet Index; hs-CRP, High-Sensitivity C-Reactive Protein; IHD, Ischemic heart disease; IL-6, Interleukin-6; LDL, Low-Density Lipoprotein; MD, Mean Difference; MED, Mediterranean Diet; NHS, Nurses’ Health Study; NHS II, Nurses’ Health Study II; OR, Odds Ratio; PDI, Plant-Based Diet Index; PREDIM, Prediabetes Risk Prediction Model; RCT, Randomized Controlled Trial; RD, Risk Difference; RR, Relative Risk; SCFA, Short-Chain Fatty Acids; SHBG, Sex Hormone-Binding Globulin; SMD, Standardized Mean Difference; SSB, Sugar-Sweetened Beverages; SR, Systematic Review; SUCRA, Surface Under the Cumulative Ranking Curve; T2D, Type 2 Diabetes Mellitus; TC, Total Cholesterol; TG, Triglycerides; uPDI, unhealthy Plant-Based Diet Index; UK, United Kingdom; WHI, Women’s Health Initiative; WMD, Weighted Mean Difference. ↑ indicates an increase; ↓ indicates a decrease. Created by the authors as a secondary synthesis of the cited literature.

## Data Availability

No new data were created or analyzed in this study. Data sharing is not applicable to this article.

## Abbreviations

The following abbreviations are used in this manuscript:

ADA	American Diabetes Association
AHEI	Alternative Healthy Eating Index
AI	Artificial intelligence
BMI	Body Mass Index
BP	Blood Pressure
CHD	Coronary Heart Disease
CI	Confidence Interval
CVD	Cardiovascular Disease
DASH	Dietary Approaches to Stop Hypertension
EASD	European Association for the Study of Diabetes
ES	Effect Size
EVOO	Extra Virgin Olive Oil
FPG	Fasting Plasma Glucose
GLP-1	Glucagon-Like Peptide-1
GRADE	Grading of Recommendations Assessment, Development and Evaluation
HbA_1c_	Glycated Hemoglobin A1c
HDL	High-Density Lipoprotein
HOMA-IR	Homeostatic Model Assessment for Insulin Resistance
HPFS	Health Professionals Follow-up Study
HR	Hazard Ratio
hPDI	Healthy Plant-Based Diet Index
hs-CRP	High-Sensitivity C-Reactive Protein
IHD	Ischemic heart disease
IDF	International Diabetes Federation
IL-6	Interleukin-6
LDL	Low-Density Lipoprotein
MD	Mean Difference
MED	Mediterranean Diet
NHS	Nurses’ Health Study
NHS II	Nurses’ Health Study II
NOVA	NOVA Food Classification System
OR	Odds Ratio
PDI	Plant-Based Diet Index
PHD	Planetary Health Diet
PREDIM	Prediabetes Risk Prediction Model
PREDIMED	Prevención con Dieta Mediterránea
RCT	Randomized Controlled Trial
RR	Relative Risk
SCFA	Short-Chain Fatty Acids
SHBG	Sex Hormone-Binding Globulin
SMD	Standardized Mean Difference
SSB	Sugar-Sweetened Beverages
SUCRA	Surface Under the Cumulative Ranking Curve
T2D	Type 2 Diabetes Mellitus
TC	Total Cholesterol
TG	Triglycerides
UK	United Kingdom
uPDI	unhealthy Plant-Based Diet Index
UPF	Ultra-Processed Food
WHI	Women’s Health Initiative
WMD	Weighted Mean Difference
